# CoFormerSurv: Collaborative transformer for multi-omics survival analysis

**DOI:** 10.1371/journal.pcbi.1013875

**Published:** 2026-01-07

**Authors:** Gang Wen, Limin Li

**Affiliations:** 1 School of Mathematics and Statistics, Zhengzhou University, Zhengzhou, Henan Province, China; 2 Institute of Mathematics, Henan Academy of Sciences, Zhengzhou, Henan Province, China; 3 School of Mathematics and Statistics, Xi’an Jiaotong University, Xi’an, Shaanxi Province, China; University of North Texas, UNITED STATES OF AMERICA

## Abstract

In the field of biomedicine, advances in high-throughput sequencing have generated vast amounts of high-dimensional multi-omics data. Survival analysis methods with multi-omics data can comprehensively uncover the heterogeneity and complexity of diseases from multiple perspectives, thereby improving prognostic predictions for patients, which is critical for developing personalized treatment strategies in precision medicine. Recently, Transformer architecture has emerged as a dominant paradigm in multiple domains. However, due to the inherent challenges in modeling right-censored data, it remains unclear how to effectively utilize Transformer architecture in multi-omics survival analysis to fully extract complementary information across different omics for improving survival prediction performance. In this work, we propose an innovative collaborative Transformer framework for multi-omics survival analysis, namely CoFormerSurv, with two consecutive Transformer architectures including an inter-omics Transformer and an inter-sample graph Transformer. The inter-omics Transformer learns multiple meaningful feature interactions by multi-head self-attention mechanism to capture and quantify complementary information across different omics, while the inter-sample graph Transformer integrates structural information from the fused multi-omics graph into the Transformer architecture, enabling more effective exploration of neighborhood relationships among samples. The two kinds of Transformer architectures can work collaboratively to generate more comprehensive multi-omics features for improving the Cox-PH model performance in survival analysis. Experimental results on multiple real-world datasets show that our proposed method outperforms both single-Transformer architectures and existing survival prediction models by simultaneously exploring complementary information from inter-omics and cross-sample perspectives.

## Author summary

We propose CoFormerSurv, a collaborative Transformer framework that improves survival prediction with multi-omics data. CoFormerSurv method consists of two complementary components: an inter-omics Transformer that models cross-omics interactions, and an inter-sample graph Transformer that learns the neighborhood relationships among multi-omics samples. By integrating these two perspectives, our dual Transformer architecture enables more comprehensive feature learning and superior performance compared to existing approaches.

## Introduction

Survival analysis uses multiple explanatory variables to predict the time until an event occurs and has always been a hot topic in the field of biomedical research. The major challenges [1] in survival analysis are the highly skewed nature of time-to-event data, in which some events occur significantly beyond the median, and censoring, which arises from lost follow-up or the termination of the study before the event of interest is observed. Clinically, survival analysis for cancer patients remains predominantly reliant on low-dimensional features (e.g., demographic factors such as age and sex, and tumor characteristics such as histologic grade and T/N/M stage) [2] to examine the effects of multiple predictors on survival outcomes. With the rapid advancement of high-throughput technology, the growing volumes of multi-omics data, such as gene expression and microRNA expression profiles, are increasingly being leveraged to predict clinical outcomes of patients. This provides new technical support and research perspectives for conducting more precise personalized prognostic prediction studies.

In traditional survival analysis, the nonparametric Kaplan-Meier (K-M) estimator, the semi-parametric Cox proportional hazards (Cox-PH) model, and the parametric accelerated failure time (AFT) model constitute three fundamental analytical approaches [3]. The K-M estimator directly estimates the survival function from survival data without making assumptions about the distribution of survival times or the form of the hazard function, but it cannot analyze or quantify the effects of explanatory variables on survival time. The Cox-PH model assumes constant hazard ratios between patients and requires no specific assumptions about the survival time distribution, which offers exceptional flexibility and broad applicability for analyzing complex survival data. The AFT model, compared to the Cox-PH model, relies on regression analysis to model covariate effects on log-survival time—a process that inherently requires distributional assumptions (e.g., Weibull or log-normal) [4]. In addition to the three aforementioned methods, traditional machine learning approaches have been successively introduced for time-to-event data analysis, such as Random Forests [5] and Support Vector Regression [6]. Recently, deep learning [7,8] has gained increasing prominence in survival analysis [9–12] due to its exceptional performance. For example, reference [10] combines deep neural networks with the AFT model to propose the deep survival analysis method DeepAFT. In contrast to DeepAFT method, DSM method [12] learns multiple latent survival distributions through hierarchical graphical models. By integrating these latent survival distributions with weighted approaches, DSM method can better accommodate the heterogeneity of survival data.

In the field of biomedicine, advances in high-throughput sequencing have generated vast amounts of high-dimensional omics data. Omics-based survival analysis methods can reveal the impact of molecular characteristics [13,14] on cancer prognosis, elucidating tumor mechanisms at the gene and pathway levels to provide evidence for personalized precision therapy. These methods can be broadly categorized into single-omics [15–17] and multi-omics [18,19] survival analysis methods. Single-omics survival analysis focuses on investigating the association between specific biomarkers (such as genomic data, transcriptomic data, or proteomic data) and patient prognosis. For example, Cox-nnet method [16] analyzes the relationship between the features of hidden layer nodes in neural networks and patient survival risk, uncovering key genes and biological pathways that significantly impact cancer prognosis, thereby revealing rich biological information. Considering the challenge of overfitting in deep learning models trained on limited high-dimensional gene expression data, reference [20] proposed VAECox, a two-stage transfer learning model that first pre-trains a VAE on multi-cancer RNA-seq data without survival labels and then transfers the learned weights to initialize a cancer-specific survival prediction model. Recently, Graph Convolutional Networks (GCNs) have achieved remarkable success in various fields, including survival prediction, due to their ability to integrate both node attributes and graph structure information. For example, AGGSurv method [21] first generates diverse sparse graph structures by randomly sampling feature subsets from high-dimensional RNA-seq data and then learns a ridge regression-Cox model to integrate predictions from these multiple GCNs, ultimately improving survival prediction performance.

Multi-omics survival analysis method integrates information from different sources to help analyze the heterogeneity and complexity of diseases from multiple dimensions, enabling more accurate predictions of patient outcomes, which is significant for advancing precision medicine. Multi-omics survival analysis methods can be divided into two categories: feature fusion-based methods [22,23] and graph fusion-based methods [24]. Feature fusion-based methods effectively capture complementary information across multiple omics by learning inter-omics feature interactions, significantly improving predictive performance for patient survival outcomes. For example, HFBSurv method [23] deeply mines omics-specific information to explore intra-omics feature dependencies and cross-omics information to quantify inter-omics feature interactions through omics-specific and cross-omics attentional factorized bilinear modules, thereby enhancing the accuracy of survival predictions. Compared to feature fusion strategies, graph fusion-based methods tend to share and propagate complementary neighborhood information across different omics. For example, GCGCN method [24] can more accurately reveal neighborhood relationships among samples for survival analysis by integrating multiple sample similarity matrices from different omics [25]. Building upon this, reference [26] proposed FGCNSurv method, which simultaneously fuses features and graphs within GCNs, enabling more comprehensive exploration of complementary relationships among multi-omics data for survival prediction.

Recently, Transformer architecture [27,28] has emerged as a dominant paradigm in multiple domains. While Transformer architecture has demonstrated promising results in survival analysis [29–31], it remains unclear how to effectively utilize Transformer architecture in multi-omics survival analysis to fully extract complementary information across different omics for improving survival prediction performance. In this work, we propose a collaborative Transformer framework for multi-omics survival analysis, namely CoFormerSurv, including two complementary Transformer architectures: an inter-omics Transformer and an inter-sample graph Transformer. The inter-omics Transformer learns multiple meaningful cross-omics features by multi-head self-attention mechanism. The inter-sample graph Transformer encodes the spatial information of the fused graph from multiple omics into the Transformer architecture to more effectively model neighborhood relations among multi-omics samples. By integrating the inter-omics Transformer and inter-sample graph Transformer, the collaborative Transformer can generate more informative and discriminative multi-omics features for Cox-PH-based survival analysis. Evaluations on multiple real-world datasets demonstrate that our proposed collaborative Transformer outperforms both single-Transformer architectures and existing survival prediction methods by jointly leveraging inter-omics and inter-sample perspectives.

## Results

### Overview of CoFormerSurv

Multi-omics survival data are typically represented as $\{x_{i}=[x_{i}^{\left(1\right)},\cdots ,x_{i}^{\left(v\right)}],O_{i},\Delta_{i}{\}}_{i=1}^{n}$, where *x*_*i*_ denotes multi-omics features of the *i*-th patient from *v* distinct omics (e.g., gene expression and microRNA expression), *O*_*i*_ signifies the observed time-to-event and $\Delta_{i}$ is a binary indicator of censoring. The observed time *O*_*i*_ depends on whether the event of death occurs before censoring. $\Delta_{i}=1$ indicates that death occurred prior to censoring, with the observed time *O*_*i*_ representing the true survival time *T*_*i*_. Conversely, $\Delta_{i}=0$ implies that *O*_*i*_ is equal to the censoring time *C*_*i*_, meaning the *i*-th patient’s follow-up ended without an observed death.

**Fig 1:**
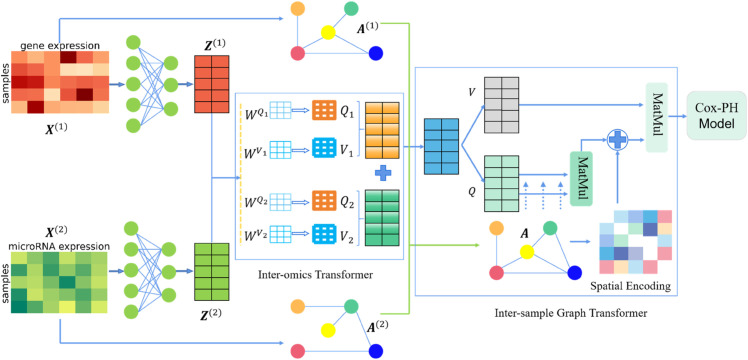
Architectural overview of the proposed CoFormerSurv model. The inter-omics Transformer employs multi-head self-attention mechanism to identify high-order interactive features across multi-omics data. The inter-sample graph Transformer encodes structural information of the fused graph from multiple omics into the Transformer architecture to aggregate multi-omics features extracted from the inter-omics Transformer for learning more expressive sample embeddings. The collaborative transformer integrates the inter-omics Transformer and the inter-sample graph Transformer to generate more informative and discriminative multi-omics features for survival analysis with the Cox proportional hazards model.

Multi-omics survival analysis methods, compared to single-omics approaches, enable more comprehensive characterization of the intricate nature and diversity of diseases from multiple perspectives, demonstrating superior potential for survival prediction. Though current multi-omics survival methods can achieve excellent performance by fusing information from different sources, it remains unclear how to effectively utilize Transformer architecture, which has become a dominant choice in many domains, to fully exploit cross-omics complementary information for multi-omics survival analysis. To tackle this issue, we propose a collaborative Transformer framework for multi-omics survival analysis, namely CoFormerSurv. CoFormerSurv method primarily includes three key components: an inter-omics Transformer, an inter-sample graph Transformer, and a Cox proportional hazards model, with the two kinds of Transformer architectures forming our collaborative Transformer framework. The inter-omics Transformer employs multi-head self-attention mechanism to identify high-order interaction features across multi-omics data. The inter-sample graph Transformer encodes structural information of the fused graph from multiple omics into the Transformer architecture to more effectively explore neighborhood relations among multi-omics samples. By aggregating interaction features extracted from the inter-omics Transformer with neighborhood relations learned from the inter-sample graph Transformer, the collaborative Transformer can generate more informative and discriminative multi-omics features for survival analysis with the Cox proportional hazards model. Fig 1 depicts the overall structure of our CoFormerSurv model. We provide a detailed description of CoFormerSurv method in the materials and methods section.

### Datasets and preprocessing

The Cancer Genome Atlas (TCGA) [32] is a comprehensive public resource that offers multi-omics data from over 11,000 patients across 33 cancer types. In this study, we evaluate the performance of CoFormerSurv method based on RNASeq-derived gene expression and miRNA-Seq-derived microRNA expression data from eight common cancer types. These eight cancer types from TCGA—including breast invasive carcinoma (BRCA), lung adenocarcinoma (LUAD), urothelial bladder carcinoma (BLCA), head and neck squamous cell carcinoma (HNSC), liver hepatocellular carcinoma (LIHC), uterine corpus endometrial carcinoma (UCEC), ovarian serous cystadenocarcinoma (OV) and lung squamous cell carcinoma (LUSC)-provide relatively sufficient sample sizes, which facilitates more reliable model training and validation. The preprocessing pipeline for gene expression and microRNA expression data from the above cancer datasets involves the following steps: First, gene/microRNA features with missing values exceeding 10% are removed, and the remaining missing values are imputed using the median strategy; Next, gene/microRNA expression levels undergo log transformation, followed by the removal of low-variance noise features; Finally, standardization is performed to normalize each gene/microRNA feature, ensuring a mean of 0 and a standard deviation of 1. After the above processing, each final cancer dataset comprises 6000 gene features and 600 miRNA features.

We further demonstrate the effectiveness of CoFormerSurv method for multi-omics survival analysis tasks on the Pan-Cancer Analysis of Whole Genomes (PCAWG) dataset. The PCAWG dataset, which comprises whole-genome sequencing data from 2,658 cancer patients collected through the International Cancer Genome Consortium (ICGC) and TCGA projects and encompasses 38 distinct tumor types, is available at https://xena.ucsc.edu/. For consistency, we applied the same preprocessing pipeline to both gene expression and microRNA expression data from the PCAWG dataset.

### Evaluation metrics

In this study, we systematically evaluated the survival prediction performance of CoFormerSurv method using two key metrics: the concordance index (C-index) and the area under the receiver operating characteristic curve (AUC). As a core evaluation metric in survival analysis, the C-index effectively measures the degree of agreement between model predictions and actual clinical observations. This metric operates on the principle that for any pair of patients, if the individual with longer actual survival time also receives a correspondingly longer predicted survival time, the prediction is considered consistent. It should be specifically noted that paired samples will be excluded from calculation under either of the following two circumstances: when the data of the patient with shorter survival time is censored, or when both patients’ data are censored observations. The C-index is mathematically defined by the following formula:

$$\text{C-index}=Prob\{\beta^{T}z_{i}^{h}>\beta^{T}z_{j}^{h}|O_{j}>O_{i},\Delta_{i}=1\},$$ (1)

Another evaluation metric, AUC, effectively assesses the accuracy of risk ranking at different event time points, with its mathematical expression being:

$$\text{AUC}=\frac{1}{N_{num}}\sum_{t\in Y}\sum_{i:O_{i}<t}\sum_{j:O_{j}>t}I(\beta^{T}z_{i}^{h}>\beta^{T}z_{j}^{h}),$$ (2)

where *Y* represents the set of observed event time points in the study data, *N*_*num*_ is the total number of all valid comparable sample pairs and I(·) is a binary indicator function. The C-index and AUC values are both bounded between 0 and 1 where 0.5 indicates predictive performance equivalent to random chance, and 1 represents perfect discrimination. The higher the values of these two metrics, the stronger the predictive ability of the model.

### Experimental setting

To comprehensively assess the performance of the proposed CoFormerSurv method, we uses a repeated holdout cross-validation strategy. The specific implementation process is as follows: First, the dataset is divided into two subsets: 80% designated for training and 20% for testing. Subsequently, a predictive model is constructed based on the training data, and the performance of the model is assessed on the testing set by calculating the C-index and AUC values. To ensure robust validation, this evaluation procedure is systematically replicated 50 times using independent random partitions, with final performance reported as mean values along with their standard deviations. Note that the training process was carried out using the Adam optimization algorithm, an adaptive stochastic gradient descent method, with a fixed learning rate of 2e-4. And during the training of CoFormerSurv model, we used cross-validation to determine the neighborhood size *K* for the graphs $G_{A^{\left(1\right)}}$ and $G_{A^{\left(2\right)}}$.

### Performance comparison with existing methods

To evaluate the performance of CoFormerSurv method in multi-omics survival analysis, we conducted comprehensive comparisons with existing state-of-the-art methods, including single-omics approaches (RSF [5], DeepSurv [9], DeepHit [11], and AGGSurv [21]) and multi-omics approaches (HFBSurv [23], SurvCNN [33], GCGCN [24], and FGCNSurv [26]). To further validate the effectiveness of our collaborative Transformer framework in extracting complementary information from multi-omics data, we developed GANMOSurv and SATMOSurv methods based on Graph Attention Network [34](GAN) and Structure-Aware Graph Transformer [35] (SAT), respectively, for patient survival prediction. Specifically, GANMOSurv and SATMOSurv methods employ graph attention network and structure-aware graph Transformer to extract features from single-omics data, and integrate these features from different omics through a neural network to learn semantically rich multi-omics representations. Tables 1 and 2 display the C-index and AUC values of various methods on the TCGA and PCAWG datasets. The upper section of each table displays the results obtained from single-omics methods on gene/microRNA expression data, while the lower section presents the corresponding results of multi-omics methods. The last columns of Tables 1 and 2 summarize the average C-index and AUC values, respectively, for each method across all cancer datasets.

**Table 1:** Comparison of the C-index performance of CoFormerSurv method with existing methods. The upper section displays the C-index values obtained from single-omics methods on gene/microRNA expression data, while the lower section presents the corresponding results of multi-omics methods. The rightmost column presents an overview of the performance, showing the average C-index value for each method across all cancer datasets.

Method	BRCA	LUAD	BLCA	HNSC	UCEC	OV	LIHC	LUSC	PCAWG	Ave
RSF	0.593/0.626	0.582/0.575	0.607/0.586	0.587/0.562	0.633/0.636	0.535/0.557	0.627/0.604	0.514/0.516	0.640/0.650	0.591/0.590
DeepSurv	0.674/0.635	0.612/0.625	0.661/0.652	0.620/0.604	0.681/0.668	0.595/0.579	0.687/0.648	0.554/0.561	0.710/0.725	0.643/0.638
DeepHit	0.696/0.623	0.622/0.612	0.657/0.651	0.618/0.606	0.689/0.655	0.601/0.577	0.698/0.657	0.557/0.563	0.713/0.709	0.650/0.628
AGGSurv	0.682/0.640	0.620/0.627	0.663/0.658	0.616/0.610	0.683/0.677	0.600/0.589	0.691/0.657	0.558/0.563	0.712/0.722	0.647/0.638
HFBSurv	0.730±0.050	0.616±0.038	0.663±0.042	0.652±0.031	0.696±0.056	0.609±0.040	0.680±0.048	0.564±0.041	0.694±0.041	0.656
SurvCNN	0.651±0.053	0.627±0.036	0.624±0.040	0.608±0.035	0.683±0.043	0.590±0.041	0.657±0.051	0.562±0.037	0.644±0.042	0.627
GCGCN	0.704±0.063	0.615±0.040	0.671±0.040	0.641±0.032	0.693±0.060	0.601±0.037	0.678±0.048	0.563±0.047	0.719±0.039	0.654
GANMOSurv	0.702±0.055	0.630±0.037	0.665±0.035	0.616±0.040	0.699±0.055	0.610±0.039	0.678±0.038	0.578±0.040	0.723±0.040	0.656
SATMOSurv	0.716±0.054	0.630±0.036	0.668±0.042	0.630±0.033	0.697±0.053	0.603±0.034	0.691±0.051	0.589±0.039	0.724±0.037	0.661
FGCNSurv	0.740±0.051	0.631±0.039	**0.686±0.035**	0.653±0.034	0.708±0.059	0.617±0.037	0.700±0.046	0.578±0.042	0.725±0.044	0.670
CoFormerSurv	**0.741±0.050**	**0.644±0.039**	**0.686±0.041**	**0.661±0.028**	**0.713±0.055**	**0.625±0.031**	**0.714±0.046**	**0.597±0.044**	**0.737±0.039**	**0.679**

The optimal and suboptimal results are highlighted in bold and underlined, respectively.

**Table 2:** The AUC values of CoFormerSurv method and existing methods. The upper section displays the AUC values obtained from single-omics methods on gene/microRNA expression data, while the lower section presents the corresponding results of multi-omics methods. The average AUC achieved by each method across all cancer datasets is summarized in the final column.

Method	BRCA	LUAD	BLCA	HNSC	UCEC	OV	LIHC	LUSC	PCAWG	Ave
RSF	0.587/0.640	0.588/0.513	0.611/0.594	0.578/0.507	0.694/0.724	0.533/0.557	0.654/0.654	0.534/0.505	0.683/0.680	0.607/0.597
DeepSurv	0.687/0.643	0.637/0.662	0.687/0.672	0.623/0.602	0.712/0.705	0.604/0.585	0.744/0.671	0.567/0.550	0.742/0.763	0.667/0.656
DeepHit	0.711/0.632	0.650/0.654	0.679/0.666	0.626/0.602	0.713/0.683	0.609/0.583	0.744/0.672	0.563/0.553	0.747/0.745	0.671/0.643
AGGSurv	0.699/0.648	0.648/0.666	0.691/0.677	0.615/0.610	0.712/0.713	0.616/0.601	0.751/0.680	0.571/0.553	0.746/0.759	0.672/0.656
HFBSurv	0.756±0.065	0.647±0.051	0.697±0.057	0.672±0.044	0.731±0.064	0.627±0.056	0.735±0.060	0.575±0.056	0.731±0.049	0.686
SurvCNN	0.654±0.063	0.654±0.048	0.643±0.061	0.615±0.050	0.717±0.051	0.602±0.061	0.696±0.065	0.566±0.054	0.678±0.046	0.647
GCGCN	0.720±0.076	0.641±0.050	0.696±0.056	0.648±0.045	0.724±0.068	0.614±0.053	0.730±0.058	0.572±0.062	0.754±0.046	0.678
GANMOSurv	0.726±0.063	0.663±0.048	0.695±0.050	0.614±0.060	0.729±0.062	0.638±0.051	0.728±0.054	0.590±0.050	0.765±0.046	0.684
SATMOSurv	0.736±0.066	0.665±0.048	0.689±0.059	0.632±0.053	0.737±0.061	0.623±0.049	0.743±0.065	0.604±0.049	0.760±0.040	0.689
FGCNSurv	0.761±0.059	0.663±0.049	**0.723±0.047**	0.665±0.049	0.747±0.064	0.638±0.052	0.756±0.054	0.590±0.051	0.757±0.051	0.701
CoFormerSurv	**0.769±0.063**	**0.677±0.051**	0.718±0.061	**0.684±0.052**	**0.752±0.061**	**0.652±0.049**	**0.773±0.054**	**0.615±0.057**	**0.781±0.046**	**0.713**

The optimal and suboptimal results are highlighted in bold and underlined, respectively.

From the results, we observe that deep learning-based survival analysis methods exhibit markedly superior performance to traditional machine learning approaches. As an illustration, the average C-index value of DeepSurv method on gene/microRNA expression data across all cancer datasets is 0.643/0.638, representing an improvement of 8.8%/8.1% compared to RSF method. Meanwhile, it is of note that multi-omics survival analysis methods (except SurvCNN) achieve more satisfactory performance than single-omics approaches in terms of C-index and AUC values, which strongly demonstrates the feasibility and effectiveness of multi-omics data integration for survival prediction. In SurvCNN method, gene and microRNA expression data are converted into an image format, allowing feature extraction via CNNs to predict the survival distribution of cancer patients. We consider that the transformation implicitly assumes the relationships among genes/microRNAs can be characterized by the spatial proximity in the images, which may not adequately capture the complex feature relationships present in the original data. Furthermore, GANMOSurv and SATMOSurv methods, which are based on graph attention mechanisms, provide performance comparable or superior to HFBSurv method by integrating multi-omics data with a simple network. Moreover, FGCNSurv method can learn more comprehensive multi-omics feature representations by dually fused graph convolutional network, outperforming GANMOSurv, SATMOSurv and GCGCN methods. More importantly, CoFormerSurv method achieves superior performance compared to all other methods. Especially for LUAD and LIHC datasets, CoFormerSurv method has a significant improvement over the suboptimal FGCNSurv method. For example, CoFormerSurv method achieves mean C-index values of 0.644 and 0.714 on LUAD and LIHC datasets, respectively, surpassing FGCNSurv method by 2.1% and 2%. These results demonstrate that CoFormerSurv method could effectively utilize the collaborative Transformer to comprehensively extract complementary information across different omics for improving multi-omics survival analysis.

**Fig 2:**
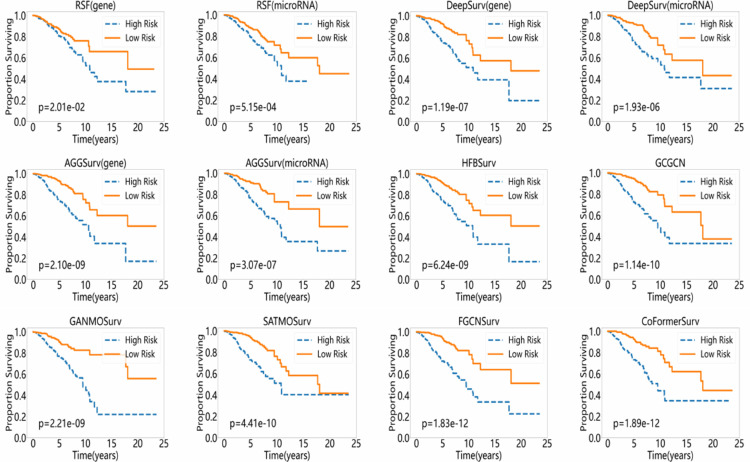
Kaplan–Meier survival curves for high-risk and low-risk groups predicted by various methods with Cox-PH model on BRCA dataset, with corresponding log-rank test p-values.

To further evaluate the performance of CoFormerSurv method, we introduced Kaplan-Meier (K-M) curves to validate its effectiveness in risk stratification of cancer patients. Specifically, we first stratified the BRCA test set samples into high-risk and low-risk groups based on the model’s predictions. Subsequently, we plotted Kaplan-Meier survival curves and performed a log-rank test to analyze the statistical differences in survival distributions between the two groups. The more significant difference in survival curves between the risk groups, the stronger the model’s discriminative ability in risk stratification. Fig 2 presents the Kaplan-Meier survival curves for the high-risk and low-risk groups predicted by various Cox-PH model-based methods on BRCA dataset, with corresponding log-rank test p-values. The results suggest that deep learning-based survival analysis methods exhibit superior discriminative capability in stratifying low-risk and high-risk groups compared to traditional machine learning approaches. For example, the log-rank test p-values generated by DeepSurv method (1.19e-07 for gene expression and 1.93e-06 for microRNA expression) demonstrated more significant statistical differences compared to those obtained by RSF method (2.01e-02 and 5.15e-04, respectively). Moreover, compared to single-omics approaches, multi-omics methods yield statistically more significant analytical results. Furthermore, in the comparison among multi-omics methods, FGCNSurv and CoFormerSurv methods achieve the best and second-best performance, respectively, at an extremely significant statistical level (1.83e-12 and 1.89e-12), significantly outperforming HFBSurv, GCGCN, GANMOSurv and SATMOSurv methods. This demonstrates that CoFormerSurv, with collaborative Transformer architecture, and FGCNSurv, with dually fused graph convolutional network, can fully leverage the complementary information across different omics data, thereby efficiently identifying high- and low-risk patient groups with highly significant survival differences.

### Ablation study

We conducted ablation studies across all cancer datasets to identify the sources of performance improvements, thereby validating the effectiveness of the collaborative Transformer architecture in multi-omics survival analysis tasks. We constructed the following model variants by ablating key components to systematically analyze each module’s impact on model performance:

- SGT: using single-omics features $Z^{\left(1\right)}/Z^{\left(2\right)}$ and graph $G_{A^{\left(1\right)}}/G_{A^{\left(2\right)}}$ instead of multi-omics features *Z^c^* and graph *G*_*A*_ to construct the inter-sample graph Transformer for learning more expressive single-omics features in survival analysis;
- MGT: directly combining single-omics features *Z*^(1)^ and *Z*^(2)^ instead of multi-omics combinatorial features *Z^c^* to construct the inter-sample graph Transformer for learning more expressive multi-omics features in survival analysis, without the inter-omics Transformer module;
- CrossT: using the combinatorial features *Z*^(*c*)^ from the inter-omics Transformer for survival analysis, without the inter-sample graph module;
- CrossT-SEGCN: using GCN with spatial encoding to aggregate multi-omics features extracted from the inter-omics Transformer for learning more expressive sample embeddings, without self-attention module in inter-sample graph Transformer;
- CrossT-SA: using self-attention mechanism to aggregate multi-omics features from the inter-omics Transformer for learning more expressive sample embeddings, without incorporating the topological structure of the multi-omics fused graph in inter-sample graph Transformer.

Table 3 reports the C-index values of CoFormerSurv method and its variants obtained by ablating key components on all cancer datasets. In this table, SGT^(1)^ and SGT^(2)^ represent SGT methods on gene expression data and microRNA expression data, respectively. The last column summarizes the average C-index values of CoFormerSurv model and corresponding variants from ablation studies across all cancer datasets. From the results presented in Table 3, we can observe that MGT (multi-omics inter-sample graph Transformer) method outperforms SGT (single-omics inter-sample graph Transformer) method on gene/microRNA expression data, which clearly demonstrates the effectiveness of multi-omics data integration. Meanwhile, these results suggest that CoFormerSurv method is superior to its variants, MGT and CrossT. Specifically, CoFormerSurv method achieves an average C-index value of 0.679 across all cancer datasets, corresponding to performance gains of 1.49% and 1.04% compared to MGT and CrossT, respectively. This improvement highlights the critical role of the inter-omics Transformer and inter-sample graph Transformer in enhancing the performance of CoFormerSurv model. Moreover, it is of note that CoFormerSurv method exhibit better performance than CrossT-SEGCN and CrossT-SA. Compared to these two variants, CoFormerSurv method can more accurately capture inter-sample neighborhood relationships by incorporating the structural information of the multi-omics fused graph into the self-attention mechanism to aggregate interaction features from the inter-omics Transformer and thereby learn more expressive embeddings. In summary, CoFormerSurv method can effectively aggregate interaction features extracted from the inter-omics Transformer with neighborhood relations learned from the inter-sample graph Transformer in the collaborative Transformer framework, to generate more informative and discriminative multi-omics features for survival analysis.

**Table 3:** The C-index values of CoFormerSurv method and its variants obtained by ablating key components. SGT^(1)^ and SGT^(2)^ represent SGT methods on gene expression data and microRNA expression data, respectively. The last column summarizes the average C-index values of CoFormerSurv model and corresponding variants across all cancer datasets.

Method	BRCA	LUAD	BLCA	HNSC	UCEC	OV	LIHC	LUSC	PCAWG	Ave
SGT^(1)^	0.722±0.062	0.627±0.043	0.668±0.038	0.644±0.033	0.699±0.056	0.622±0.037	0.700±0.048	0.572±0.042	0.717±0.042	0.664
SGT^(2)^	0.637±0.056	0.628±0.044	0.651±0.041	0.606±0.034	0.673±0.061	0.583±0.041	0.649±0.053	0.566±0.041	0.714±0.040	0.634
MGT	0.730±0.062	0.632±0.039	0.681±0.035	0.656±0.033	0.702±0.056	0.613±0.033	0.705±0.047	0.580±0.041	0.727±0.039	0.669
CrossT	0.731±0.051	0.634±0.045	0.681±0.037	0.656±0.029	0.707±0.056	0.621±0.034	0.710±0.046	0.587±0.044	0.725±0.041	0.672
CrossT-SEGCN	0.727±0.051	0.625±0.040	0.674±0.036	0.642±0.031	0.701±0.051	0.617±0.036	0.704±0.044	0.578±0.045	0.730±0.040	0.668
CrossT-SA	0.736±0.054	0.640±0.038	**0.691±0.038**	0.647±0.031	0.699±0.052	0.622±0.031	0.700±0.047	0.575±0.041	0.729±0.041	0.671
CoFormerSurv	**0.741±0.050**	**0.644±0.039**	0.686±0.041	**0.661±0.028**	**0.713±0.055**	**0.625±0.031**	**0.714±0.046**	**0.597±0.044**	**0.737+0.039**	**0.679**

The optimal and suboptimal results are highlighted in bold and underlined, respectively.

### Feature analysis

**Fig 3:**
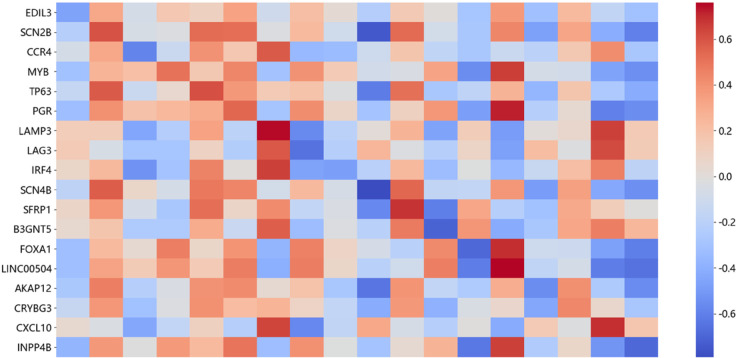
Pearson correlation coefficients between the expression levels of the identified genes and the feature representations from each hidden node in the output layer of the feature extraction network.

We further investigate the hidden nodes of CoFormerSurv model to identify molecular markers that significantly impact cancer patient survival. The specific procedure for molecular marker selection is as follows: First, we computed the Pearson correlation coefficients between the raw gene expression data and the feature representations from each hidden node in the output layer of the feature extraction network. Subsequently, we selected the top-ranked genes for each hidden node by their absolute correlation value. Fig 3 presents the Pearson correlation coefficients between feature representations at different hidden nodes and expression levels of selected gene markers in breast cancer training samples.

Many of the identified gene signatures exhibit oncogenic relevance to breast carcinogenesis and significantly correlate with patient survival outcomes. The EDIL3 gene exerts multifaceted pro-tumorigenic functions within the tumor microenvironment. The EDIL3 gene [36] is reported to enhances cellular invasion and accelerates lung metastasis by activating the integrin-FAK signaling pathway in breast cancer cells. The CCR4 gene, a key chemokine receptor involved in modulating immune homeostasis, is closely associated with tumor growth and metastasis. Research [37] has shown that CCR4 gene expression varies significantly among human breast cancer cell lines, with particularly marked overexpression in those exhibiting high metastatic potential. The LAMP3 gene encodes a lysosomal membrane protein involved in lysosomal function and autophagy-related processes. In breast cancer cell lines, LAMP3 gene [38] exhibits differential expression, with its expression levels induced in a manner dependent on oxygen concentration. FOXA1 gene [39] binds to condensed chromatin, facilitating the recruitment of other transcription factors, and plays a pivotal role in breast cancer pathogenesis. FOXA1 is also frequently mutated in ER+ breast cancer. LINC00504 gene serves as a potentially relevant lncRNA in breast oncogenesis. Patients with higher median expression of LINC00504 exhibited a higher survival probability in the basal-like subtype [40]. The B3GNT5 gene plays an essential role in synthesizing lacto- and neolacto-series glycans on glycolipids, and these carbohydrate structures are essential for embryonic development. B3GNT5 gene expression was significantly upregulated in basal-subtype breast cancer cell lines and its elevated expression levels show significant correlations with larger tumor size and poorer survival rates [41]. The SCN4B gene encodes the $\beta_{4}$ protein, which not only serves as a important regulatory subunit of sodium channels but also plays a key role in suppressing cancer metastasis. Research [42] has shown that low expression of the SCN4B gene in breast cancer biopsy tissues is significantly associated with the occurrence of high-grade primary tumors and metastatic tumors.

### Performance of CoFormerSurv method on different multi-omics data types

**Fig 4:**
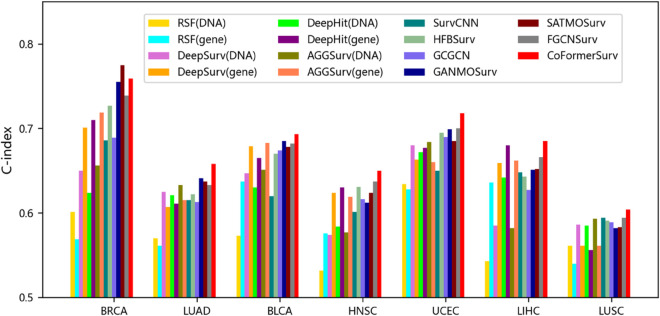
The C-index values of comparative methods with gene expression and/or DNA methylation data. RSF, DeepSurv, DeepHit, and AGGSurv are single-omics methods with gene expression or DNA methylation data. The remaining methods are multi-omics approaches, utilizing both DNA methylation and gene expression data.

**Fig 5:**
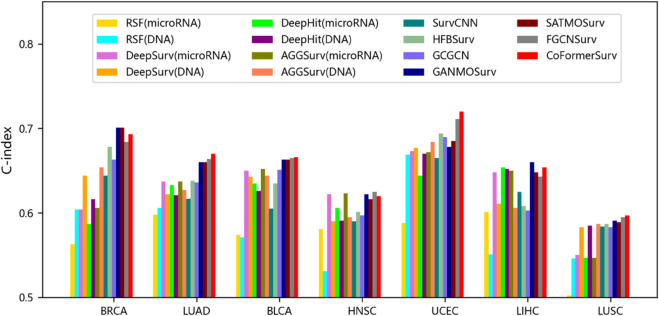
The C-index values of various methods with DNA methylation and/or microRNA expression data. RSF, DeepSurv, DeepHit, and AGGSurv are single-omics methods with DNA methylation or microRNA expression data. The remaining methods are multi-omics approaches, utilizing both DNA methylation and microRNA expression data.

We further evaluated CoFormerSurv method on different types of multi-omics data, such as gene expression coupled with DNA methylation, as well as microRNA expression with DNA methylation. We preprocessed the DNA methylation data using the same strategy mentioned above, removing noise-prone variables with low inter-sample variability to balance information retention and computational efficiency. By reducing feature dimensionality, downstream analyses can focus on the most informative biological signals while preventing overfitting. We ultimately retained the top 6000 high-variance features — a common practice in the field — which effectively balances information preservation with model robustness and is well-suited for downstream multi-omics integration analyses. Due to insufficient sample size in DNA methylation training data for ovarian cancer (OV), which could not meet the requirements for statistical analysis, this cancer type was excluded from the study. The final experiments were conducted on multi-omics datasets from seven different cancer types, with detailed evaluation results presented in Figs 4 and 5 and Tables 4 and 5.

**Table 4:** The AUC values of CoFormerSurv method and existing methods with gene expression and/or DNA methylation data. The upper section displays the AUC values from single-omics methods using either gene expression or DNA methylation data, while the lower section presents the corresponding results of multi-omics methods.

Method	BRCA	LUAD	BLCA	HNSC	UCEC	LIHC	LUSC
RSF	0.588/0.602	0.544/0.554	0.639/0.554	0.586/0.541	0.680/0.656	0.689/0.527	0.523/0.526
DeepSurv	0.715/0.653	0.622/0.648	0.706/0.671	0.631/0.570	0.676/0.685	0.714/0.601	0.575/0.595
DeepHit	0.732/0.624	0.627/0.641	0.685/0.644	0.645/0.579	0.69/0.672	0.725/0.652	0.563/0.587
AGGSurv	0.737/0.661	0.633/0.659	0.709/0.670	0.623/0.577	0.672/0.691	0.720/0.600	0.573/0.604
HFBSurv	0.738±0.086	0.641±0.064	0.704±0.053	0.643±0.050	0.711±0.087	0.695±0.059	0.600±0.057
SurvCNN	0.700±0.079	0.634±0.067	0.629±0.064	0.609±0.051	0.663±0.078	0.686±0.059	0.603±0.053
GCGCN	0.689±0.097	0.634±0.062	0.694±0.052	0.615±0.052	0.702±0.086	0.675±0.062	0.594±0.063
GANMOSurv	0.777±0.078	0.669±0.053	0.718±0.046	0.621±0.048	0.711±0.086	0.703±0.057	0.588±0.052
SATMOSurv	**0.803±0.061**	0.672±0.055	0.709±0.048	0.638±0.058	0.699±0.088	0.707±0.062	0.591±0.057
FGCNSurv	0.755±0.079	0.656±0.067	0.715±0.047	0.649±0.055	0.717±0.087	0.725±0.060	0.608±0.053
CoFormerSurv	0.775+0.081	**0.694+0.057**	**0.730+0.055**	**0.671+0.049**	**0.734+0.086**	**0.739+0.062**	**0.620+0.049**

The optimal and suboptimal results are highlighted in bold and underlined, respectively.

**Table 5:** The AUC values of CoFormerSurv method and existing methods with DNA methylation and/or microRNA expression data. The upper section displays the AUC values from single-omics methods using either DNA methylation or microRNA expression data, while the lower section presents the corresponding results of multi-omics methods.

Method	BRCA	LUAD	BLCA	HNSC	UCEC	LIHC	LUSC
RSF	0.601/0.572	0.582/0.584	0.593/0.550	0.494/0.543	0.703/0.640	0.539/0.617	0.529/0.449
DeepSurv	0.648/0.608	0.644/0.664	0.674/0.675	0.598/0.634	0.687/0.694	0.631/0.671	0.589/0.543
DeepHit	0.611/0.589	0.644/0.665	0.64/0.647	0.597/0.622	0.675/0.656	0.67/0.675	0.581/0.546
AGGSurv	0.661/0.608	0.651/0.663	0.670/0.677	0.603/**0.637**	0.696/0.689	0.625/0.674	0.596/0.539	
HFBSurv	0.685±0.092	0.661±0.064	0.666±0.047	0.606±0.054	0.715±0.086	0.635±0.070	0.597±0.067
SurvCNN	0.662±0.075	0.638±0.071	0.623±0.056	0.593±0.055	0.682±0.086	0.646±0.056	0.594±0.069
GCGCN	0.657±0.097	0.658±0.067	0.681±0.049	0.601±0.056	0.700±0.092	0.629±0.074	0.589±0.062
GANMOSurv	0.713±0.090	0.689±0.052	0.691±0.052	0.633±0.052	0.691±0.081	**0.690±0.062**	0.598±0.054
SATMOSurv	**0.719±0.087**	0.688±0.063	0.688±0.054	0.626±0.051	0.703±0.083	0.677±0.059	0.597±0.058
FGCNSurv	0.684±0.091	0.691±0.053	0.697±0.048	0.632±0.059	0.728±0.084	0.672±0.066	0.604±0.061
CoFormerSurv	0.709±0.090	**0.702±0.057**	**0.701±0.049**	0.633±0.051	**0.738±0.074**	0.686±0.049	**0.612±0.602**

The optimal and suboptimal results are highlighted in bold and underlined, respectively.

The experimental results demonstrate that multi-omics methods significantly outperform single-omics methods across various types of datasets. Specifically, in multi-omics data with gene expression and DNA methylation, FGCNSurv method achieves superior performance with an average C-index of 0.665 across all cancer datasets. This represents an improvement of 3.7% over DeepSurv method using gene expression data alone, and an improvement of 7.1% when trained solely on DNA methylation data. Notably, CoFormerSurv method exhibits exceptional performance among multi-omics methods. For example, CoFormerSurv method achieves average C-index values of 0.681 and 0.660 across all cancer datasets in different types of multi-omics data, representing improvements of 4.2% and 4.0% over HFBSurv method. Additionally, further analysis reveals notable performance variations of CoFormerSurv method across different types of multi-omics data. Compared to multi-omics data that includes DNA methylation and microRNA expression, this method demonstrates superior performance in multi-omics data that includes gene expression and DNA methylation. Specifically, the improvement of CoFormerSurv over FGCNSurv is not particularly pronounced in multi-omics data with DNA methylation and microRNA expression. In contrast, with data that includes gene expression and DNA methylation, CoFormerSurv outperforms the second-best FGCNSurv method, achieving an average C-index improvement of 2.6% across all cancer datasets. In summary, these results indicate that the CoFormerSurv method effectively leverages collaborative Transformer to comprehensively extract complementary information across different omics, thereby enhancing multi-omics survival analysis.

## Discussion

Multi-omics data encompasses multidimensional molecular information, including genes, microRNA, and DNA methylation. The integration of multi-omics data enables a systematic exploration of the interactions and functional mechanisms among biological components, providing robust technical support for cancer subtype identification, drug target discovery, and optimization of clinical decision-making systems.

Multi-omics-based survival analysis methods can more comprehensively uncover the heterogeneity and complexity of diseases, which is of significant importance for the advancement of precision medicine. In this work, we propose CoFormerSurv method, an innovative collaborative Transformer framework for multi-omics survival analysis. CoFormerSurv method includes an inter-omics Transformer, an inter-sample graph Transformer, and a Cox proportional hazards model, with the two kinds of Transformer architectures working together to form our collaborative Transformer framework. CoFormerSurv method, on one hand, identifies higher-order interaction features in multi-omics data through an inter-omics Transformer to quantify the complex relationships within the data; on the other hand, it encodes structural information from the fused multi-omics graph into the Transformer architecture via an inter-sample graph Transformer to learn more expressive sample embeddings. CoFormerSurv method can effectively aggregate interaction features extracted from the inter-omics Transformer with neighborhood relations learned from the inter-sample graph Transformer in the collaborative Transformer framework, to generate more informative and discriminative multi-omics features for survival analysis with the Cox proportional hazards model. Compared with single-Transformer approaches and state-of-the-art alternatives, our collaborative Transformer achieves superior survival prediction performance on multiple real-world datasets, by simultaneously exploring complementary information from both inter-omics and cross-sample perspective. Furthermore, the majority of key genes identified through hidden-layer feature analysis show significant correlations with cancer patient survival rates.

Although CoFormerSurv method has made significant progress in multi-omics survival analysis, there remains vast potential for its further optimization and exploration. First, the proportional hazards assumption in CoFormerSurv method often does not align with real-world scenarios. Enabling the model to directly learn the probability distribution of patient survival time may potentially yield further performance improvements. Second, CoFormerSurv method could integrate multi-modal data, such as genomic and histopathological images, to achieve a more comprehensive and accurate prediction of patient survival outcomes. Additionally, cancer subtype classification with multi-omics data has recently garnered widespread attention in the field of oncology research. In the future, we will explore how to leverage the collaborative Transformer architecture for multi-omics-based cancer subtype classification, thereby advancing the precision of molecular classification and the development of personalized medicine.

## Materials and methods

### Inter-omics transformer

The essence of multi-omics integration is to uncover cross-omics interactions to fully exploit their complementary information for downstream tasks. For example, contrastive learning [43] can identify discriminative patterns to improve survival prediction [44] by capturing notable cross-correlations across different omics. In this work, we use a Transformer architecture [27], namely inter-omics Transformer, to learn multiple meaningful feature interactions among multiple omics by multi-head self-attention mechanism. Transformer architecture with self-attention mechanism has demonstrated remarkable success across multiple domains, including computer vision tasks such as image segmentation [45] and classification [46], as well as machine translation [27] in natural language processing. The core idea of the self-attention mechanism is to dynamically learn a set of key weights that allocate importance to different parts of the input data. This operational principle exhibits intrinsic similarities with human visual cognition: when processing complex visual information, the human brain instinctively suppresses irrelevant background noise and focuses attentional resources on key areas relevant to the current task. This biomimetic attention allocation mechanism enables the model to autonomously capture the most discriminative features within the data, thereby enhancing the efficiency and accuracy of information processing.

The inter-omics Transformer enables the determination of which key features from different omics can be fused to generate meaningful combinatorial features for survival analysis. Following the architectural approach proposed in [47], we use the same matrix for keys and queries in the inter-omics Transformer architecture to reduce the number of learnable parameters. Specifically, for pre-processed gene expression data *X*^(1)^ and microRNA expression data *X*^(2)^, we first project them into a latent space through a single-layer fully-connected network, obtaining compact low-dimensional feature representations $\left\{Z^{\left(l\right)}=[z_{1}^{\left(l\right)},\cdots ,z_{n}^{\left(l\right)}{]}^{T},l\in \{1,2\}\right\}$. Subsequently, we derive query and value representations $\left\{Q^{\left(l\right)}=[q_{1}^{\left(l\right)},\cdots ,q_{n}^{\left(l\right)}{]}^{T},V^{\left(l\right)}=[v_{1}^{\left(l\right)},\cdots ,v_{n}^{\left(l\right)}{]}^{T},l\in \{1,2\}\right\}$ for gene and microRNA via learnable parameter matrices $\left\{W^{Q^{\;c}},W^{V^{\;c}}\right\}$ as below:


$$\{q_{i}^{\left(l\right)},v_{i}^{\left(l\right)}\}=\{W^{Q^{\;c}},W^{V^{\;c}}\}\cdot z_{i}^{\left(l\right)}.$$

We then compute the cross-omics feature representations $Z^{\;c}=\{Z^{\left(1,c\right)},Z^{\left(2,c\right)}\}$ through an attention-weighted aggregation of the value vectors $\left\{V^{\left(1\right)},V^{\left(2\right)}\right\}$. Specifically, for each omics type, the contextualized feature $z_{i}^{\left(u_{1},c\right)}$ is derived as:

$$z_{i}^{\left(u_{1},c\right)}=\sum_{u_{2}}{\hat{\alpha }}_{i}^{u_{1},u_{2}}v_{i}^{\left(u_{2}\right)},u_{1},u_{2}\in \{1,2\},$$ (3)

where the attention weights ${\hat{\alpha }}_{i}^{u_{1},u_{2}}$ are obtained by softmax normalization:

$${\hat{\alpha }}_{i}^{u_{1},u_{2}}=\frac{exp(\alpha_{i}^{u_{1},u_{2}})}{\sum_{u_{2}}exp(\alpha_{i}^{u_{1},u_{2}})},\alpha_{i}^{u_{1},u_{2}}=\frac{\langle q_{i}^{\left(u_{1}\right)},q_{i}^{\left(u_{2}\right)}\rangle }{\sqrt{d^{\;c}}}.$$ (4)

Here, these attention weights are computed using scaled dot-product operation, defined by the inner product ⟨·,·⟩ and scaled by *d^c^* (the dimensionality of $v_{i}^{\left(1\right)}$), effectively capturing the correlation among different omics features.

To capture diverse and meaningful feature interactions, we employ a multi-head learning strategy to independently extract distinct combinatorial features, denoted as $Z^{c_{1}}$ and $Z^{c_{2}}$. Subsequently, we use a fully connected network to compress these different combinatorial features into an information-rich multi-omics representation $Z^{\;c}=[z_{1}^{\;c},\cdots ,z_{n}^{\;c}{]}^{T}$ , formulated as:

$$Z^{\;c}=f_{1}^{\;c}(Z^{c_{1}}W_{1}^{\;c})⊕f_{1}^{\;c}(Z^{c_{2}}W_{1}^{\;c}),$$ (5)

where $W_{1}^{\;c}$ represents the learnable parameter matrix, $f_{1}^{\;c}$ is the activation function, and ⊕ denotes matrix concatenation along the feature dimension.

### Inter-sample graph transformer

Although the inter-omics Transformer effectively integrates multi-omics feature information for individual patients, it does not fully account for the potential relationship among patient populations. The inter-sample graph Transformer encodes structural information of the fused graph from multiple omics into the Transformer architecture [28,35] to more effectively explore neighborhood relations among multi-omics samples. By aggregating interaction features extracted from the inter-omics Transformer with neighborhood relations learned from the inter-sample graph Transformer, our proposed collaborative Transformer framework can learn more expressive sample embeddings for survival analysis. We first present the Transformer without encoding the graph structure and then describe how these two Transformer architectures are integrated to work collaboratively. The core attention mechanism of the Transformer is defined as:

$$Z=\text{softmax}(\frac{QQ^{T}}{\sqrt{d}})V,$$ (6)

where $Q=Z^{\;c}W^{Q}$ and $V=Z^{\;c}W^{V}$. Here $Q=[q_{1},\cdots ,q_{n}{]}^{T},V=[v_{1},\cdots ,v_{n}{]}^{T}$ denote query and value representations, $Z=[z_{1},\cdots ,z_{n}{]}^{T}$ represents multi-omics features that incorporate node-level information, *d* corresponds to the dimension of $v_{i}$, and $\left\{W^{Q},W^{V}\right\}$ are learnable parameter matrices.

The output of the Transformer is permutation-invariant in the input data and ignores the adjacency relations among samples. Traditional GCN-based approaches typically assign equal or pre-defined weights to all neighboring nodes, relying on a static aggregation mechanism that struggles to adaptively differentiate the importance variations between nodes. To address the aforementioned issues, we incorporate the topological structure of the multi-omics fused graph into the transformer architecture. This design enables the model to dynamically focus on semantically similar samples and effectively filter out irrelevant local connections, thereby overcoming the limitations of fixed aggregation schemes. We employ the following strategy to construct a fused graph from multiple omics. Firstly, given gene expression data *X*^(1)^, we construct a *K*-NN graph $G_{A^{\left(1\right)}}$ and use the exponential similarity kernel to define the adjacency matrix $A^{\left(1\right)}\in R^{n\times n}$ as follows:

$$A^{\left(1\right)}(i,j)=\left\{ \begin{array}{cc} \exp(\frac{-\rho^{2}(x_{i}^{\left(1\right)},x_{j}^{\left(1\right)})}{\mu \delta^{2}}), & j\in N_{i}^{\left(1\right)}\\ 0, & \text{otherwise}, \end{array}\right.$$ (7)

Here ρ(·,·) represents the Euclidean distance metric and $N_{i}^{\left(1\right)}$ denotes the set of *k*-nearest neighbors for patient *i*. $\delta^{2}$ is introduced to normalize pairwise distances and is empirically set as the median of all squared Euclidean distances across patients. The hyperparameter μ is configured as 0.3/0.2 for the gene/microRNA graph to adjust the scaling. Similarly, we obtain the *K*-NN graph $G_{A^{\left(2\right)}}$ based on microRNA expression data *X*^(2)^, with *A*^(2)^ representing its adjacency matrix. Then we fuse the two graphs $G_{A^{\left(2\right)}},G_{A^{\left(2\right)}}$ by taking the union of their edges and averaging of their adjacency matrices, resulting in a unified graph *G*_*A*_ with adjacent matrix *A*. To more effectively characterize the topological structure of the multi-omics fused graph, we perform spectral transformation on the adjacency matrix *A* to obtain the corresponding convolution matrix $\hat{A}$, as detailed below:

$$\hat{A}={\tilde{D}}^{-\frac{1}{2}}\tilde{A}{\tilde{D}}^{-\frac{1}{2}},$$ (8)

where $\tilde{A}=A+I_{n}$ and $\tilde{D}=diag({\tilde{d}}_{1},{\tilde{d}}_{2},\ldots ,{\tilde{d}}_{n})$ is defined as a diagonal matrix with entries with ${\tilde{d}}_{i}=\sum_{j}{\tilde{A}}_{ij}$.

Subsequently, we encode the spatial information between samples in the multi-omics fused graph *G*_*A*_ into the traditional Transformer to bias the attention scores. More precisely, we enhance the attention kernel using the corresponding convolution matrix $\hat{A}$ on the fused graph *G*_*A*_ as follows:

$$\hat{\alpha }\left(i,j\right)=\frac{\text{exp}({q_{i}}^{T}q_{j}/\sqrt{d})*{\hat{A}}_{\left(i,j\right)}}{\sum_{j^{\prime }\in N_{i}}\text{exp}({q_{i}}^{T}q_{j^{\prime }}/\sqrt{d})*{\hat{A}}_{\left(i,j^{\prime }\right)}},$$ (9)

where *N*_*i*_ denotes the set of neighboring patients for patient *i* in the fused graph *G*_*A*_. We leverage the calibrated attention weights to aggregate the value representations *V*, obtaining multi-omics features *Z* that incorporate node-level information. These features are then integrated through a fully connected network to learn more expressive representations $Z^{h}=[z_{1}^{h},\cdots ,z_{n}^{h}{]}^{T}$ of the samples, formulated as:


$$z_{i}=\sum_{j\in N_{i}}\hat{\alpha }\left(i,j\right)v_{j},$$

$$z_{i}^{h}=f_{1}(z_{i}W_{1})$$ (10)

where *W*_1_ and *f*_1_ denote the learnable parameter matrix and activation function, respectively.

### Survival analysis with Cox-PH model

Survival analysis focuses on modeling the distribution of survival time *T*. The statistical distribution characteristics of survival time *T* can be fully described by its corresponding survival function *S*(*t*) and hazard function λ(t). *S*(*t*) is defined as the probability that an individual survives beyond time *t*:


$$S(t)=P(T>t)=1-\int_{0}^{t}f(s)ds,$$

where *f*(*s*) denotes the probability density function of the survival time. The hazard function λ(t) represents the instantaneous event occurrence rate at time *t*, conditional on survival up to that time. Formally, it is defined as:


$$\lambda (t)=\lim_{\Delta t\to 0}\frac{P(t\leq T<t+\Delta t|T>t)}{\Delta t}.$$

For a specified parametric form of the survival time distribution, we can estimate the probability distribution of patients’ survival times by maximizing the complete likelihood function for both censored and uncensored observations, as follows:


$$L=\prod_{i}f(O_{i}|x_{i}{)}^{\Delta_{i}}S(O_{i}|x_{i}{)}^{1-\Delta_{i}}.$$

The Cox-PH model [48] formulates the hazard function as a multiplicative relationship between a baseline hazard and covariate effects:


$$\lambda (t|x_{i})=\lambda_{0}(t)\text{exp}(\beta^{T}x_{i}),$$

where the baseline hazard function $\lambda_{0}(t)$ has no restrictions and β denotes the coefficient vector to be estimated. Due to its flexibility and interpretability, the Cox-PH model has become one of the most widely used methods in survival analysis.

Based on the more expressive representations $Z^{h}=[z_{1}^{h},\cdots ,z_{n}^{h}{]}^{T}$, the loss of our multi-omics survival analysis with Cox-PH model is

$$-\text{log}(L_{cox})=-\sum_{i=1}\Delta_{i}[\beta^{T}z_{i}^{h}-\text{log}\sum_{j:O_{j}>O_{i}}\text{exp}(\beta^{T}z_{j}^{h})].$$ (11)

We minimize the loss function to learn the parameters of collaborative Transformer and β of the Cox-PH model. Once obtaining the parameter estimates, the risk function λ(t) based on patients’ multi-omics data can be further estimated through the Breslow estimator [49].

In summary, to construct the CoFormerSurv model for multi-omics survival analysis, we need to train four core components including feature extraction layer to generate compact, low-dimensional feature representations from single-omics data, inter-omics Transformer to identify multiple meaningful feature interactions across multi-omics data, inter-sample graph Transformer to encode the structural information of the fused graph from multiple omics into the Transformer architecture for aggregating multi-omics features extracted from the inter-omics Transformer, and Cox-PH model for the final survival analysis.

## Supporting information

S1 Text Fig A illustrates the variation in C-index values of the CoFormerSurv method with the hyperparameter *K* (the neighborhood size for graph construction). Fig B presents an overview of the overall architecture of the CoFormerSurv model for integrating three omics data types. Table A shows a comparison of the time and space complexity across different methods, including CoFormerSurv. Table B compares the C-index values of the CoFormerSurv method and existing methods with gene expression and/or copy number variation data. Table C displays the C-index values of various methods on three types of omics data including gene expression, microRNA expression and DNA methylation. Table D reports the C-index values of the CoFormerSurv method across different dimensionalities for the feature representation *z*. Tables E–F list the p-values from significance tests for the C-index and AUC of the CoFormerSurv method and existing state-of-the-art methods on gene expression and/or microRNA expression data.
(PDF)
