# Supplementary material for: CoFormerSurv: Collaborative transformer for multi-omics survival analysis
Source: PLoS Comput Biol. 2026 Jan 7;22(1):e1013875. doi: 10.1371/journal.pcbi.1013875 (PMC12890224; doi:10.1371/journal.pcbi.1013875)
Supplement: S1 Text — Fig B presents an overview of the overall architecture of the CoFormerSurv model for integrating three omics data types. Table A shows a comparison of the time and space complexity across different methods, including CoFormerSurv. Table B compares the C-index values of the CoFormerSurv method and existing methods with gene expression and/or copy number variation data. Table C displays the C-index values of various methods on three types of omics data including gene expression, microRNA expression and DNA methylation. Table D reports the C-index values of the CoFormerSurv method across different dimensionalities for the feature representation z. Tables E–F list the p-values from significance tests for the C-index and AUC of the CoFormerSurv method and existing state-of-the-art methods on gene expression and/or microRNA expression data. (PDF) [file pcbi.1013875.s001.pdf]

Supplementary Material

1 Sensitivity analysis of the neighborhood size K for graph construction

Although the hyperparameter K (the neighborhood size for graph construction) can be determined through cross-validation, to systematically assess the impact of parameter K on model performance, we plotted the variation curves of C-index values for CoFormerSurv method across different cancer datasets in Fig A, as K ranges from 2 to 15. The experimental results indicate that while the predictive performance of CoFormerSurv model exhibits minor fluctuations with variation in K values, it maintains overall stability. Notably, when K value falls within a relatively large range, CoFormerSurv model consistently demonstrates significantly better predictive performance compared to other benchmark methods. This finding suggests that CoFormerSurv method exhibits strong adaptability to K-value selection in real-world applications, with its parameter configuration showing high stability and robustness. These characteristics provide reliable technical support for clinical medical applications.

2 Complexity comparison

To assess the computational cost of CoFormerSurv method, we compare the time and space complexity of different methods. The space complexity of the model is characterized by its count of trainable parameters. Its time complexity is evaluated by the wall-clock time required to perform 100 training epochs on BRCA dataset with gene expression and microRNA expression data, using a specified hardware configuration (Intel Core i9-14900K @3.2 GHz CPU, Windows OS). As shown in Table A, on both gene expression and microRNA expression data, the training time required for DeepHit method to predict the discrete distribution of survival time significantly exceeds that required for DeepSurv method to predict the relative risk. This difference is primarily attributed to the fact that the former needs to learn a complete probability distribution, whereas the latter only outputs a relative risk score. For multi-omics methods, model training time is generally proportional to spatial complexity. Our method demonstrates a favorable bal-

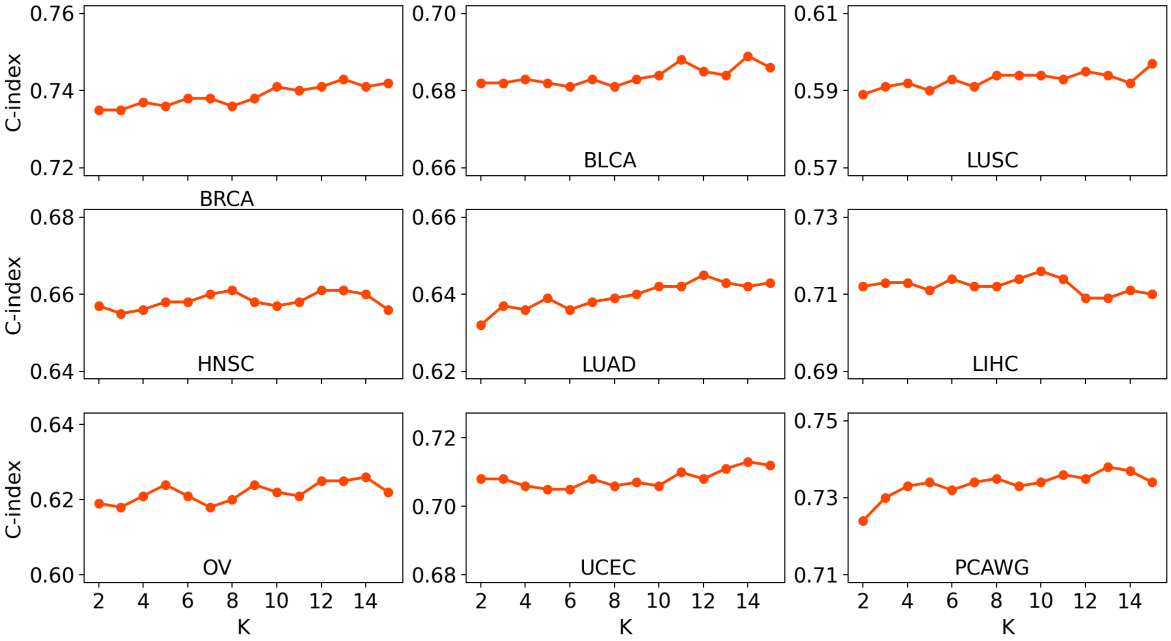

Fig A. The C-index values of CoFormerSurv method with varying hyperparameter K. CoFormerSurv method exhibits robust performance despite minor fluctuations with changes in K.

Table A. The time and space complexity of different methods.

Data	Method	Time Complexity	Space Complexity
gene/ microRNA	DeepSurv	360839.98ms/63345.27ms	23.70MB/0.50MB
	DeepHit	644633.53ms/360327.16ms	23.71MB/0.51MB
multi-omics	HFBSurv	90926.41ms	1.81 MB
	SurvCNN	181831.60ms	1.22MB
	GCGCN	645327.04ms	25.99 MB
	GANMOSurv	339945.36ms	2.64MB
	SATMOSurv	506695.03ms	2.79MB
	FGCNSurv	91646.20ms	1.43MB
	CoFormerSurv	135678.21ms	2.76MB

ance between efficiency and performance. Specifically, it is faster and requires less storage than methods such as GCGCN, GANMOSurv, and SATMOSurv. Although more computationally intensive than HFBSurv and FGCNSurv, the significant performance improvement achieved by our method fully justifies the additional computational cost.

3 Performance of CoFormerSurv method on multi-omics data comprising gene expression and copy number variation

To evaluate the performance of CoFormerSurv method, we compared CoFormerSurv method with existing state-of-the-art methods on multi-omics data comprising gene expression and copy number variation (CNV). We sourced copy number variation (CNV) data for eight common and high-incidence cancer types from UCSC Xena database (https://xena.ucsc.edu/). The data were preprocessed by removing noise-prone variables with low inter-sample variability and retaining the top 6,000 high-variance variables for downstream modeling. This is a standard practice in high-dimensional omics analysis to balance information retention with computational efficiency, thereby ensuring that the subsequent integrated analysis focuses on the most informative biological signals while effectively mitigating overfitting. Table B presents the C-index values of CoFormerSurv method and existing methods with gene expression and/or copy number variation data. The experimental results indicate that the single-omics methods based on copy number variation perform poorly. Nevertheless, several multi-omics methods—including HFBSurv, SATMOSurv, FGCNSurv, and the proposed CoFormerSurv—demonstrate improvements over their single-omics counterparts. However, in the face of high-dimensional and information-scarce data, these improvements remain modest, with some methods failing to show a clear advantage. In contrast, the proposed CoFormerSurv method achieves performance that is significantly superior to all comparative methods. In summary, by effectively integrating an inter-omics Transformer with an inter-sample graph Transformer, our CoFormerSurv method can generate more informative and discriminative multi-omics features for survival analysis.

Table B. The C-index values of CoFormerSurv method and existing methods with gene expression and/or copy number variation data.

Data	Method	BRCA	BLCA	LUAD	LUSC	HNSC	LIHC	UCEC	OV
gene	RSF	0.590	0.598	0.563	0.528	0.583	0.612	0.610	0.554
	DeepSurv	0.683	0.663	0.606	0.552	0.623	0.684	0.677	0.602
	DeepHit	0.705	0.653	0.611	0.544	0.629	<u>0.699</u>	0.679	0.606
	AGGSurv	0.696	0.666	0.613	0.555	0.624	0.688	0.679	0.610
CNV	RSF	0.584	0.468	0.512	0.523	0.532	0.498	0.465	0.511
	DeepSurv	0.564	0.552	0.561	0.541	0.542	0.570	0.624	0.582
	DeepHit	0.568	0.574	0.550	0.544	0.540	0.565	0.622	0.590
	AGGSurv	0.566	0.553	0.565	0.542	0.542	0.571	0.628	0.578
multi-omics	HFBSurv	<u>0.733</u>	0.655	0.593	0.562	0.638	0.664	<u>0.702</u>	0.617
	SurvCNN	0.626	0.625	0.610	0.553	0.598	0.620	0.664	0.588
	GCGCN	0.711	0.639	0.599	0.568	0.623	0.650	0.689	0.615
	GANMOSurv	0.703	0.664	<u>0.622</u>	0.554	0.607	0.681	0.683	0.606
	SATMOSurv	0.705	**0.671**	**0.624**	0.560	0.614	0.671	0.679	<u>0.622</u>
	FGCNSurv	0.728	0.645	0.613	<u>0.570</u>	<u>0.637</u>	**0.700**	<u>0.702</u>	0.615
	CoFormerSurv	**0.737**	<u>0.670</u>	0.621	**0.571**	**0.648**	0.690	**0.709**	**0.627**

The optimal and suboptimal results are highlighted in bold and underlined, respectively.

4 Evaluation of CoFormerSurv method on three types of omics data including gene expression, microRNA expression and DNA methylation

We integrated three omics data types—including gene expression, microRNA expression, and DNA methylation—to further validate the performance of CoFormerSurv method. The structure of CoFormerSurv model with three omics data types is illustrated in Fig B. The model employs two distinct inter-omics Transformer modules: one for gene expression and microRNA expression data, and the other for gene expression and DNA methylation data, to capture diverse inter-omics features. A single-layer network then consolidates these inter-omics features into a dense, information-rich representation. Simultaneously, an inter-sample graph Transformer

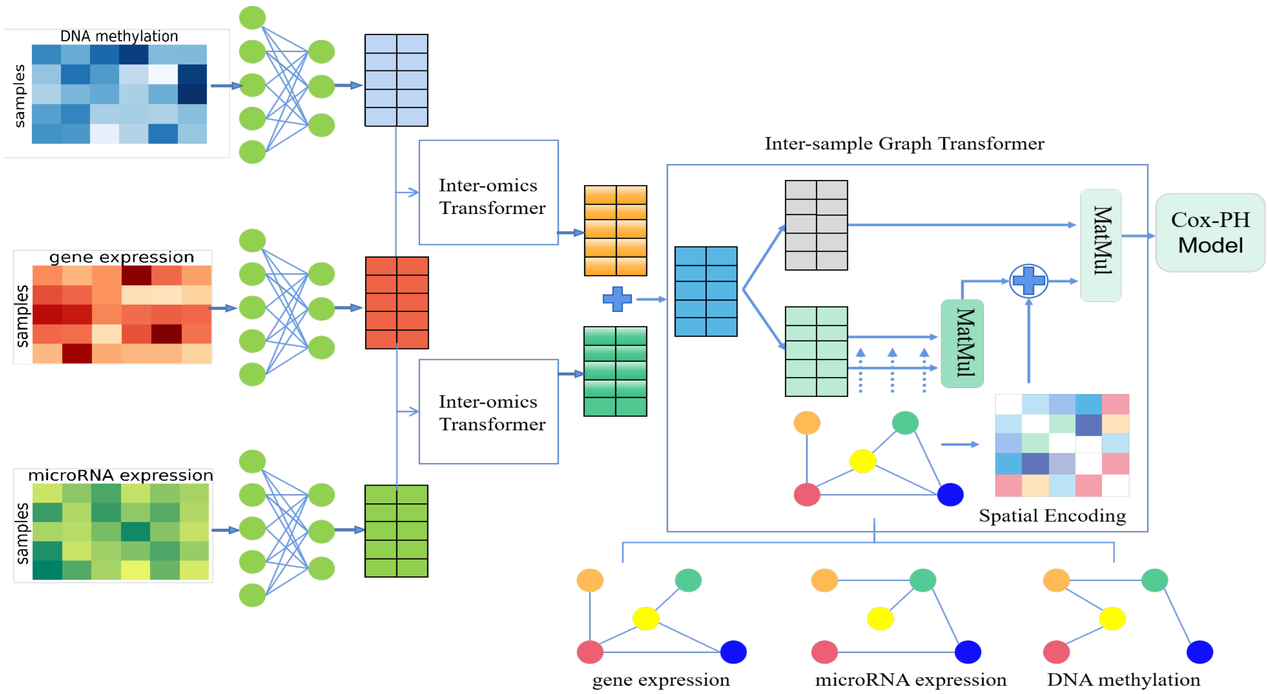

Fig B. Architectural overview of the proposed CoFormerSurv model with three omics data types. The model employs two distinct inter-omics Transformer modules: one for gene expression and microRNA expression data, and the other for gene expression and DNA methylation data, to capture diverse inter-omics features. Simultaneously, an inter-sample graph Transformer encodes the structural information of the fused graph from three omics into the Transformer architecture, to more effectively model neighborhood relations among multi-omics samples. By integrating both the inter-omics and inter-sample graph Transformers, the collaborative Transformer produces highly discriminative multi-omics representations for Cox-PH-based survival analysis.

encodes the structural information of the fused graph from three omics into the Transformer architecture, enabling more effective exploration of neighborhood relationships among samples. By integrating both the inter-omics and inter-sample graph Transformers, the resulting collaborative Transformer produces highly discriminative multi-omics representations for survival analysis based on the Cox-PH model. Table C displays the C-index values of various meth-

Table C. Comparision of the C-index performance of CoFormerSurv method with existing methods on three types of omics data including gene expression, microRNA expression and DNA methylation.

Data	Method	BRCA	BLCA	LUAD	LUSC	HNSC	LIHC	UCEC
gene	RSF	0.609	0.607	0.607	0.513	0.587	0.627	0.616
	DeepSurv	0.699	0.661	0.600	0.552	0.620	0.687	0.662
	DeepHit	0.708	0.657	0.611	0.560	0.618	0.698	0.672
	AGGSurv	0.717	0.663	0.607	0.551	0.618	0.691	0.658
DNA methylation	RSF	0.646	0.558	0.565	0.546	0.548	0.550	0.617
	DeepSurv	0.654	0.648	0.618	0.583	0.575	0.607	0.680
	DeepHit	0.629	0.635	0.619	0.585	0.580	0.642	0.669
	AGGSurv	0.664	0.650	0.623	0.587	0.582	0.605	0.684
microRNA	RSF	0.600	0.586	0.582	0.502	0.562	0.604	0.589
	DeepSurv	0.610	0.655	0.632	0.550	0.609	0.656	0.676
	DeepHit	0.591	0.651	0.627	0.547	0.606	0.657	0.650
	AGGSurv	0.612	0.658	0.633	0.547	0.610	0.657	0.676
multi-omics	HFBSurv	0.731	0.673	0.614	0.582	0.630	0.672	0.686
	SurvCNN	0.684	0.640	0.608	<u>0.594</u>	0.598	0.659	0.661
	GCGCN	0.698	0.669	0.608	0.588	0.613	0.655	0.690
	GANMOSurv	0.747	0.660	0.631	0.578	0.612	0.681	0.677
	SATMOSurv	<u>0.752</u>	0.674	<u>0.637</u>	0.585	0.632	0.687	0.684
	FGCNSurv	0.745	<u>0.685</u>	0.626	0.581	<u>0.646</u>	<u>0.703</u>	<u>0.698</u>
	CoFormerSurv	**0.760**	**0.693**	**0.654**	**0.607**	**0.655**	**0.710**	**0.714**

The optimal and suboptimal results are highlighted in bold and underlined, respectively.

ods on three types of omics data including gene expression, microRNA expression and DNA methylation. As shown in Table C, most multi-omics methods demonstrate superior predictive performance compared to those using any single omics data type (gene expression, microRNA expression, or DNA methylation), with FGCNSurv method and CoFormerSurv method showing particularly outstanding results. This indicates that CoFormerSurv method with collaborative Transformer architecture and FGCNSurv method with dually fused graph convolutional net-

work can effectively leverage complementary information across different omics data to enhance cancer survival prediction. Notably, CoFormerSurv method with three omics data types significantly outperforms the second-best method FGCNSurv, further validating the effectiveness and superiority of the collaborative Transformer architecture.

5 Sensitivity analysis for the dimensionality of the multi-omics feature

To investigate the sensitivity of CoFormerSurv to the dimensionality of the multi-omics feature z, we compared the model's performance when z was set to dimensions of 60, 80, and 100. Table D presents the C-index values of CoFormerSurv method for feature representations z of different dimensionalities. As shown in Table D, CoFormerSurv method exhibits strong robustness, showing minimal sensitivity to the dimension of the multi-omics feature z despite performance variations across different cancer datasets.

Table D. Performance comparison of CoFormerSurv on different dimensions of the embedding representation using C-index values.

Dim	BRCA	BLCA	LUAD	LUSC	HNSC	LIHC	UCEC	OV	PCAWG	Ave
60	0.743	0.685	0.640	0.597	0.661	0.709	0.713	0.624	0.736	0.678
80	0.739	0.684	0.642	0.595	0.662	0.716	0.712	0.620	0.735	0.679
100	0.741	0.686	0.644	0.597	0.661	0.714	0.713	0.625	0.737	0.679

6 Statistical significance test for the performance improvement of CoFormerSurv

We conducted t-tests to demonstrate the superiority of CoFormerSurv method over existing state-of-the-art methods. T-test uses the t-distribution to evaluate whether an observed difference between two groups of data samples is genuine, based on the probability (p-value) of that difference arising by chance. A smaller p-value indicates a lower likelihood that the results are due to random variation, thereby providing stronger evidence of a statistically significant

difference. In Tables E-F, we reported the p-values from significance tests for the C-index and AUC of CoFormerSurv method and existing state-of-the-art methods on gene expression and/or microRNA expression data. The results show that the performance improvement of CoFormerSurv method over existing methods has widespread statistical significance. A further notable trend is that the magnitude of improvement is associated with heightened statistical significance. These results further demonstrate that CoFormerSurv method could effectively utilize the collaborative Transformer to comprehensively extract complementary information across different omics for improving multi-omics survival analysis.

Table E. Statistical significance tests for C-index values of CoFormerSurv method and existing methods on gene expression and/or microRNA expression data.

Data	gene/microRNA				multi-omics
Method	RSF	DeepSurv	DeepHit	AGGSurv	HFBSurv
BRCA	5.17e-22/2.15e-15	3.31e-07/1.25e-14	1.61e-04/5.42e-16	5.76e-06/4.20e-14	2.95e-01
LUAD	2.13e-07/2.68e-21	3.18e-04/4.66e-01	1.30e-02/3.48e-02	4.57e-03/2.87e-01	4.33e-03
BLCA	2.64e-14/1.08e-19	4.09e-03/4.94e-05	1.04e-03/3.87e-05	6.78e-03/6.77e-04	6.99e-03
HNSC	5.58e-17/1.58e-21	1.61e-07/4.71e-11	3.87e-08/5.19e-12	5.54e-10/8.62e-11	1.45e-01
UCEC	3.66e-11/1.47e-08	6.83e-03/7.01e-04	3.85e-02/9.42e-07	6.10e-03/9.27e-04	1.16e-01
OV	6.80e-19/3.88e-11	1.77e-05/1.95e-07	4.69e-04/4.75e-09	6.77e-05/1.07e-06	3.05e-02
LIHC	4.13e-14/6.98e-19	8.43e-03/6.58e-09	1.22e-01/1.88e-07	1.04e-02/1.35e-08	6.38e-04
LUSC	5.89e-13/5.83e-12	4.03e-05/2.26e-04	1.10e-04/2.14e-04	2.54e-05/4.45e-04	6.87e-04
PCAWG	4.24e-20/3.89e-18	6.68e-04/1.50e-01	2.63e-03/8.27e-04	5.21e-04/5.16e-02	9.77e-07
Data	multi-omics				
Method	SurvCNN	GCGCN	GANMOSurv	SATMOSurv	FGCNSurv
BRCA	2.64e-13	2.09e-03	4.56e-04	1.96e-02	9.12e-01
LUAD	2.50e-02	7.35e-04	6.11e-02	5.69e-02	8.05e-02
BLCA	1.91e-11	7.29e-02	7.64e-03	3.01e-02	9.56e-01
HNSC	2.01e-12	1.84e-03	6.79e-09	6.34e-06	2.21e-01
UCEC	3.02e-03	8.69e-02	1.92e-01	1.43e-01	6.40e-01
OV	9.71e-06	6.00e-04	3.16e-02	1.40e-03	2.49e-01
LIHC	1.07e-07	3.32e-04	7.80e-05	2.30e-02	1.27e-01
LUSC	1.35e-04	9.70e-04	4.80e-02	2.54e-01	6.50e-02
PCAWG	2.92e-19	2.33e-02	8.88e-02	1.08e-01	1.53e-01

Table F. Statistical significance tests for AUC values of CoFormerSurv method and existing methods on gene expression and/or microRNA expression data.

Data	gene/microRNA				multi-omics
Method	RSF	DeepSurv	DeepHit	AGGSurv	HFBSurv
BRCA	6.57e-24/6.74e-14	1.55e-07/1.79e-14	5.85e-05/4.22e-16	2.47e-06/3.19e-14	2.68e-01
LUAD	2.47e-10/1.17e-13	2.43e-04/8.57e-02	1.25e-02/1.82e-04	1.31e-03/4.83e-02	5.54e-04
BLCA	5.26e-10/2.18e-09	1.14e-02/1.79e-04	2.10e-03/5.37e-05	2.51e-02/7.44e-04	8.07e-02
HNSC	1.92e-08/1.12e-16	6.24e-07/5.07e-10	6.63e-07/4.18e-12	5.68e-09/6.60e-10	2.07e-01
UCEC	1.42e-04/4.06e-02	2.92e-03/5.86e-04	4.44e-03/2.31e-07	2.62e-03/1.44e-03	1.01e-01
OV	5.31e-11/2.62e-09	1.10e-05/1.64e-06	1.87e-05/1.27e-08	2.70e-05/9.04e-06	2.08e-02
LIHC	8.67e-10/4.06e-09	1.49e-02/1.98e-12	1.61e-02/1.76e-12	2.65e-02/6.12e-12	1.79e-03
LUSC	4.59e-07/8.35e-11	1.62e-04/1.97e-08	1.98e-05/2.24e-08	1.59e-04/2.34e-08	6.67e-04
PCAWG	3.01e-16/1.48e-17	2.33e-05/5.31e-02	1.68e-04/1.48e-04	2.27e-05/1.59e-02	7.95e-07
Data	multi-omics				
Method	SurvCNN	GCGCN	GANMOSurv	SATMOSurv	FGCNSurv
BRCA	3.62e-15	6.12e-04	7.26e-04	1.06e-02	4.78e-01
LUAD	3.06e-02	5.36e-04	4.01e-02	6.19e-02	4.36e-02
BLCA	2.56e-08	6.50e-02	4.20e-02	1.85e-02	6.70e-01
HNSC	1.03e-09	3.10e-04	1.58e-08	2.49e-06	5.63e-02
UCEC	2.98e-03	3.95e-02	6.69e-02	2.27e-01	6.81e-01
OV	2.12e-05	3.71e-04	1.78e-01	4.03e-03	5.17e-03
LIHC	6.31e-09	2.36e-04	1.11e-04	1.61e-02	1.21e-01
LUSC	2.82e-05	5.47e-04	2.21e-02	2.83e-01	2.71e-02
PCAWG	3.55e-19	4.05e-03	8.33e-02	1.55e-02	1.75e-02
